# Functional Validation of a Constitutive Autonomous Silencer Element

**DOI:** 10.1371/journal.pone.0124588

**Published:** 2015-04-24

**Authors:** Heyuan Qi, Mingdong Liu, David W. Emery, George Stamatoyannopoulos

**Affiliations:** 1 CAS Key Laboratory of Genome Sciences and Information, Beijing Institute of Genomics, Chinese Academy of Sciences, Beijing, China; 2 University of Chinese Academy of Sciences, Beijing, China; 3 Department of Medicine, Division of Medical Genetics, University of Washington, Seattle, WA, United States of America; 4 Institutional Biosafety Committee Services, WIRB-Copernicus Group, Puyallup, WA, United States of America; University of Birmingham, UNITED KINGDOM

## Abstract

Sequences of the genome that are capable of silencing gene expression are thought to play a key role in gene regulation. However, very few silencer elements capable of functioning in mammalian cells have been described, and only a fraction of these have been tested for the ability to function in an autonomous fashion. We report here the characterization and functional validation of a constitutive autonomous silencer element from the human genome called T39, and the comparison of T39 to three other putative silencer elements previously described by others. Functional analysis included one assay for enhancer-blocking insulator activity and two independent assays for silencer activity, all based on stable transfection and comparison to a neutral spacer control. In erythroid K562 cells, T39 exhibited potent silencer activity, the previously described element PRE2-S5 exhibited modest silencer activity, and the two other previously described elements exhibited no silencer activity. T39 was further found to be capable of silencing three disparate promoters, of silencing gene expression in three disparate cell lines, and of functioning as a single copy in a topology-independent manner. Of the four elements analyzed, only T39 exhibits a constitutive pattern of DNase hypersensitivity and binding by CTCF. In its native location the T39 element also exhibits a unique interaction profile with a subset of distal putative regulatory elements. Taken together, these studies validate T39 as a constitutive autonomous silencer, identify T39 as a defined control for future studies of other regulatory elements such as insulators, and provide a basic chromatin profile for one highly potent silencer element.

## Introduction

Mammalian gene expression is regulated in *cis* by DNA sequences that serve as binding sites for proteins which support or suppress transcription either by directly interacting with transcription factors or by modifying the surrounding chromatin. Information generated by the ENCODE project [1], as well as similar studies have led to the chromatin profiling and functional characterization of several classes of chromatin regulatory sequences, including enhancers, promoters, and more recently chromatin insulators [2–4]. Regulatory elements capable of silencing gene expression have also been described in several species (for example, see [5,6]). However, the number of silencers indentified in the mammalian genomes has been very limited, resulting in a dearth of information regarding silencer-specific chromatin profiles and mechanisms of action. Limited information suggests there are two general categories of silencers: elements that only function in a specific context to repress promoter activity (termed negative regulatory elements [5]); and elements that function in an autonomous, context-independent manner. Negative regulatory elements function by recruiting proteins which disrupt or inactivate the formation of functional Pol II transcription complexes at otherwise accessible promoters. This is accomplished by recruiting repressor proteins, blocking the nearby binding of activator proteins, or competing directly with activator proteins for the same binding site (reviewed in [6]). Autonomous silencers function by establishing a repressive chromatin state that can be stably inherited [7]. This is typically accomplished by recruiting proteins capable of modifying DNA (e.g. methyltransferases) or histone tails (e.g. histone deacetylases) in a manner that supports the formation of heterochromatin, or proteins that help stabilize and propagate heterochromatin (e.g. polycomb group proteins, heterochromatin protein 1). This in turn prevents activators and transcription factors from accessing gene promoters.

The availability of a very limited number of autonomous mammalian silencer elements described in the scientific literature has made it difficult to identify common mechanisms of action, as well as common chromatin profiles. Advances toward indentifying and characterizing autonomous silencer elements have also been hampered by the lack of independently-validated functional assays. A question even remains as to whether silencer activity determined by transient transfection of reporter constructs can accurately predict whether a particular sequence is capable of providing silencer activity in the setting of intact chromatin. We report here the use of two functional assays based on stable transfection of K562 cells, which allow for the identification of potential silencers in the human genome. Using these assays we characterize a novel silencer element from the human genome, silencer T39, which is active in mammalian cells. We also use these stable transfection assays to functionally characterize elements previously reported to function as silencers on the basis of transient assays. Finally, we present data indicating that the T39 silencer is functional in combination with three distinct promoters and in three distinct cell lines, and that it physically interacts with other putative regulatory elements within its native locus.

## Materials and Methods

### Cell lines

The following human cell lines were used for these studies: K562 (bone marrow, myelo/erythro-leukemia, ATCC CCL-243), HEK-293 (embryonic kidney, ATCC CRL-1573), HeLa (epithelial, adenocarcinoma, ATCC CCL-2), and HepG2 (liver, hepatocellular carcinoma, ATCC HB-8065). K562 cells were cultured in Iscove's Modified Dulbecco's Medium supplemented with 10% fetal bovine serum (IMDM/FBS) and the other cell lines were cultured in Dulbecco's Modified Eagles Medium supplemented with 10% fetal bovine serum (DMEM/FBS) at 37°C, 5% CO_2_.

### Drug-resistant colony assay

The drug-resistant colony assay has been described previously [4,8,9]. The reporter plasmids contain an expression cassette for the G418-resistance gene *neo*, transcribed by a promoter from the human γ -globin gene HBG1 and terminated with a polyadenylation sequence from the virus SV40. High level reporter gene expression is dependent on an enhancer derived from DNase I hypersensitive site 2 (HS2) of the mouse β-globin gene cluster and placed 3′ of the *neo* expression cassette. In order to assess both silencer and enhancer-blocking insulator activity, candidate sequences are inserted immediately 5′ of the *neo* expression cassette and 3′ between the *neo* expression cassette and HS2 enhancer. In order to assess silencer activity only, candidate sequences are inserted immediately 5′ of the *neo* expression cassette and 3′ of the HS2 enhancer. Candidate sequences were inserted by MultiSite Gateway exchange [9]. Reporter plasmids were linearized and transfected into K562 cells at a dose of 2 μg per 10^6^ cells using the Amaxa Biosystems Nucleofector II electroporator and Nucleofector V kit (Lonza Group Ltd., Basel, Switzerland). The cells were then resuspended in 5 mL IMDM/FBS and cultured for 2 days to allow for genomic integration of the plasmid. After this, the cells were collected, counted, resuspended at a dose of 5 x10^5^ cells in 5 mL IMDM, 20% FBS, 0.8% low-melting agarose, and 1 mg/mL of the neomycin drug analog G418 (active component), and plated in 60 mm tissue culture dishes in triplicate. These semisolid cultures were incubated another 2–3 weeks and then scored for colony formation. Inserts included the PRE2-S5, MECP2 F3, PDGFA 5′SHS, and T39 putative silencers listed in Table 1, the 1.2 kb version of the prototypic chromatin insulator cHS4 element as a positive insulator control [8], and a 308 bp fragment from the bacterial drug resistance gene *zeo* as a neutral spacer control [10]. See S1 Table for details on datasets.

**Table 1:** Putative silencers reported in the literature.

Element	Genomic Coordinates	Size (bp)	Assay	Enhancer / Promoter	Reporter Topology^(a)^	Control^(a)^	Cells	Effect	Ref.
PRE2-S5	Human hg19 chr11:69378896–69379848	953	Transient Luciferase	PRE1/ CCND1	P→R-E-S	No E No S	MCF7	50-fold	[11]
Gal4-CBX4	Yeast 9xGal4 binding site	153	Transient Luciferase	none/CMV	S-I-P→R	With I	HEK293	2-fold	[16]
MECP2F3	Human hg19 chrX:153357647–153358631	985	Transient Luciferase	SV40/SV40	S-P→R-E	No S	SK-N-SH, HT1080 CRL1718, HeLa	10-fold	[12]
Myh6 PNR	Rat rn5 chr15:37516688–37516719	32	Transient CAT	none/Myh6	P-S→R S-P→R	No S	Cardiomyocytes, HeLa, NIH3T3, Jeg	20-fold	[17]
TSHB 3'	Human hg19 chr1:115571965–115572317	353	Transient CAT	none/TSHB	S-P→R	No S	HeLa, HEK293 GH3, TtT-97	15-fold	[18]
OVAL NRE	Chicken galGal4 chr2:67779445–67779665	221	Transient CAT	none/OVAL none/Tk	S-P→R P→R-S	No S	Primary oviduct cells	8-fold 4-fold	[19]
PDGFA 5' SHS	Human hg19 chr7:560885–560915	31	Transient Luc / CAT	none/PDGFA none/Tk	S-P→R P→R-S	No S	BSC-1, HepG2 U87	2-fold 5-fold	[13]
T39	hg19 chrX:11551258–11551578	321	Stable G418^r^ colony	HS2/HBG1	S-P→R-E-S	Spacer	K562	23-fold	[4]

^(a)^ P→R, Promoter transcribing Reporter gene; S, silencer; E, enhancer, I, barrier insulator.

### GFP reporter assay

The GFP reporter plasmids contain an expression cassette for GFP terminated with a polyadenylation sequence from the virus SV40. This cassette is transcribed either by the same HBG1 promoter used for the drug-resistance colony assay reporter plasmid, the promoter from cytomegelovirus (CMV), or the promoter from the human PGK gene. These reporter constructs were either used directly or with the T39 silencer element inserted either 5′ or 3' of the expression cassette. Reporter plasmids were either left circularized or were linearized with Sca I, and transfected into HEK293 cells, K-562 cells, HeLa cells, or HepG2 cells as for the *neo* colony assay, except that the cells were expanded in DMEM/FBS for 7 days without selection. The cells were then collected, washed, and analyzed by flow cytometry for reporter GFP expression. The level of expression was determined by measuring the mean fluorescence of the cells located within the GFP-positive gate. See S1 Table for details on datasets.

### Informatics

Data involving chromatin profiles, including CTCF binding, Digital DNase footprinting, DNase I hypersensitivity, and CTCF Interactions, where derived from data publically available on the UCSC Genome Browser (http://genome.ucsc.edu).

Track names used for analysis include the following:

CTCF binding: K562 CTCF TFS ChIP-seq raw signal rep 1/2 from ENCODE/UW.
Digital DNase I footprinting: K562 DNase I DGF per-base signal rep 1/2 from ENCODE/UW.
DNase I hypersensitivity: K562 DNase I HS raw signal rep 1/2 from ENCODE/UW.
CTCF interactions: K562 CTCF ChIA-PET Interactions rep 1/2 form ENCODE/GIS.

## Results

### Experimental Approach

**Fig 1:**
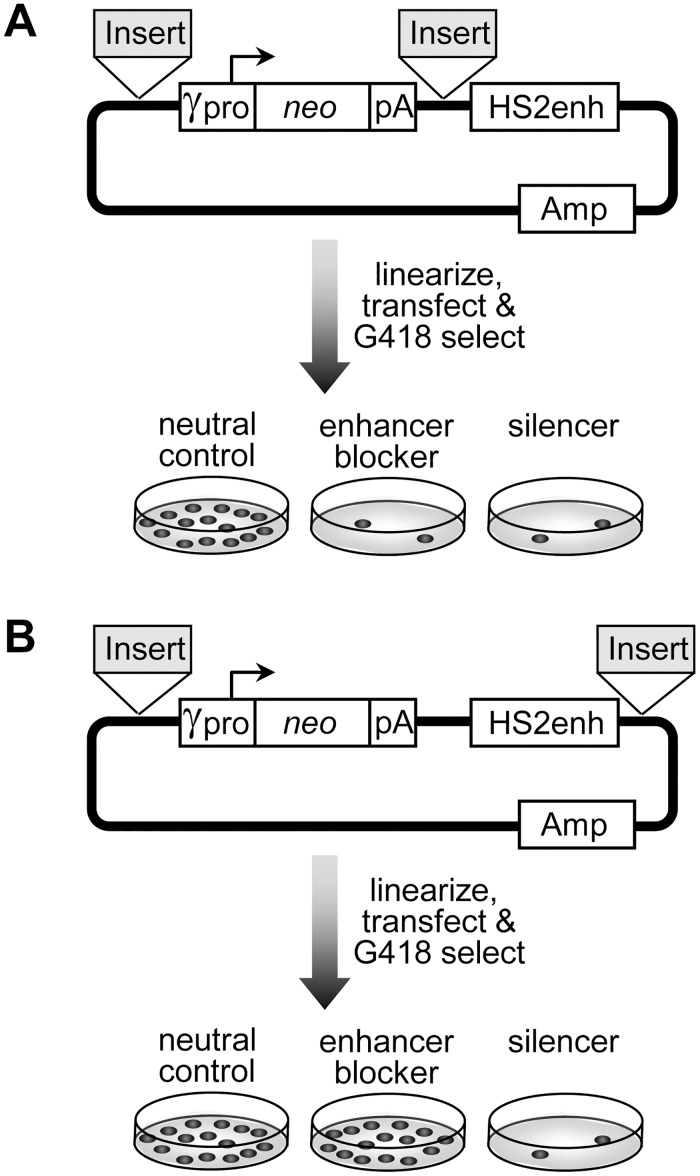
Schema for drug-resistant colony assay. (A) Enhancer-blocking and silencer assay. The reporter plasmid used for this assay contains an expression cassette for the bacterial drug resistance gene *neo* transcribed by an erythroid promoter from the γ-globin gene HBG1 and terminated with the SV40 polyadenylation signal. An erythroid HS2 enhancer from the β-globin locus control region is located 3′ of this cassette. Candidate elements are inserted 5′ of the expression cassette and 3′ between the expression cassette and the enhancer. After being linearized, the plasmid is transfected into erythroid K562 cells, which are then analyzed for the frequency of colony formation under selection with the neomycin drug analog G418. Both enhancer-blocking insulator and silencers are expected to reduce the rate of G418-resistant colony formation. (B) Silencer-only assay. The reporter plasmid is the same as for the enhancer-blocking assay, except that candidate elements are inserted 5′ of the *neo* expression cassette and 3′ of the HS2 enhancer in a manner that brackets both elements and allows the enhancer to interact with the promoter. As above, the plasmids are linearized and transfected into K562 cells, which are then analyzed for the frequency of drug-resistant colony formation. In this case, only silencers are expected to reduce the rate of G418-resistant colony formation.

In our search for enhancer-blocking insulators from the human genome, we used reporter constructs in which candidate sequences were placed between an expression cassette for the drug-resistance gene *neo* and a potent enhancer (Fig 1A) as described previously [4,8,9]. If the insert contains an enhancer blocking insulator, it will disrupt communication between the enhancer and promoter, resulting in a decreased level of *neo* gene expression and a decreased rate of colony formation when transfected K562 cells are grown in a semisolid culture in the presence of neomycin drug analog G418. However, this assay cannot distinguish between enhancer-blocking insulators and elements capable of silencing gene expression, since both activities cause a decrease in *neo* gene expression. To distinguish between these two activities, it is necessary to use a second assay in which the candidate element is instead used to bracket the enhancer and *neo* gene expression cassette from the outside (Fig 1B) as described previously [4,8]. In this configuration, enhancer-blocking insulators are no longer capable of blocking enhancer-promoter interactions and thus have no effect on *neo* gene expression or the rate of drug-resistant colony formation. In contrast, elements capable of silencing gene expression should still be capable of reducing *neo* gene expression in this setting, resulting in a reduction in the rate of drug-resistant colony formation.

### Initial characterization of the T39 silencer

**Fig 2:**
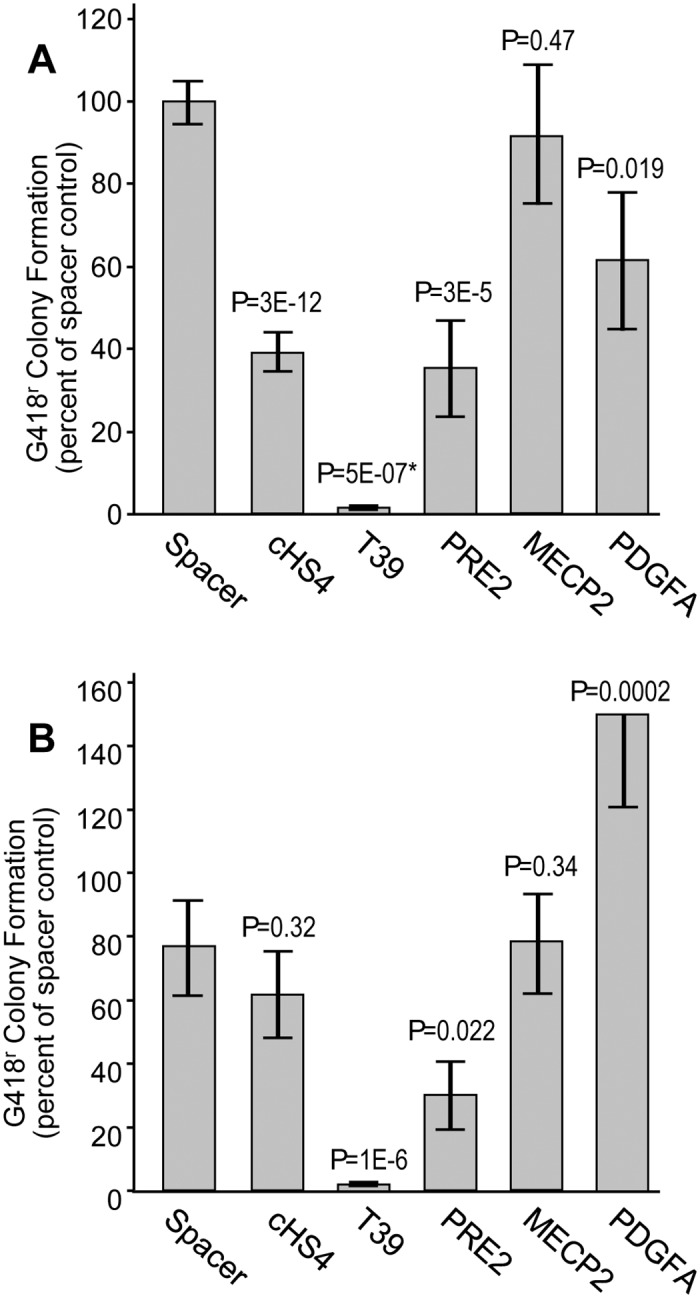
Assessment of silencer activity by drug-resistance colony formation. (A) Assessment of putative silencer elements in the enhancer-blocking insulator assay. The indicated candidates were inserted into the enhancer-blocking insulator reporter construct and analyzed for G418-resistant colony formation in K-562 cells. (B) Assessment of putative silencer elements in the silencer assay. The indicated candidates were inserted into the silencer reporter construct and analyzed for G418-resistant colony formation in K-562 cells. Data are shown for the mean ± s.e. from two or more independent transfections plated in triplicate, and are normalized the colony formation achieved with the spacer control. Spacer, 308 bp fragment from the bacterial gene zeo; cHS4, 1.2 kb fragment containing the cHS4 chromatin insulator. P values are based on one-sided *t*-test compared to the spacer control, except for the T39 sample in panel A. As indicated by (*), in this one case the data did not follow a normal distribution determined by the Lilliefors test, so the P value is based on the Wilcoxon rank-sum test compared to the spacer control. See S1 Table for details on datasets.

Based on previous studies [4], we expanded our search for enhancer-blocking insulators to include sequences from the human genome that contain multiple CTCF binding motifs located within 50 to 200 bp from each other. In addition, we required that these motifs belong to classes with over 50% probability of being occupied by CTCF as determined by ChIP-seq as previously described [4]. One of the sequences we tested, designated T39, was found to exhibit very strong activity in the enhancer-blocking assay. In the experiments shown in Fig 2A, the frequency of colony formation for the insulator assay construct containing T39 was only 2% of that for the neutral spacer control, compared to 40% for the prototypic enhancer-blocking chromatin insulator cHS4. However, as seen in Fig 2B, the T39 element also decreased the rate of colony formation to 2% of that for the neutral spacer control in the silencer assay. In comparison, the cHS4 insulator had no statistically distinguishable effect on colony formation in this assay. Taken together, these studies demonstrate that the T39 element is a powerful silencer element.

**Fig 3:**
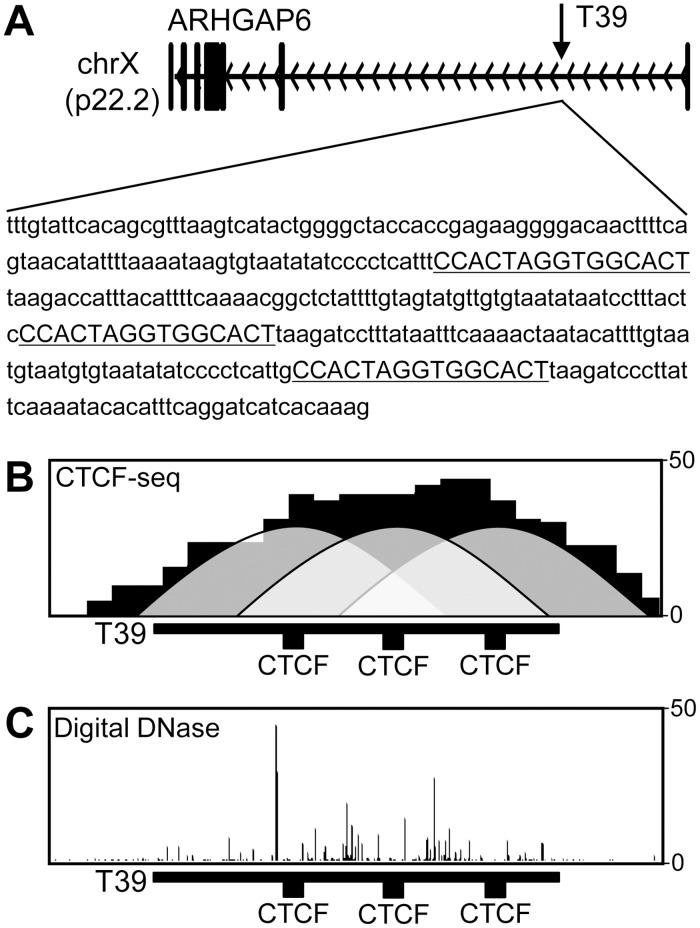
Genomic properties of the native T39 locus. (A) Chromosome location. The location of the T39 element is shown within the body of the ARHGAP6 gene at p22.2 of the human X chromosome. Below this is shown the primary sequence of the T39 element used for functional studies, with the consensus sequences for the three class-38 CTCF-binding motifs indicated in underlined bold type face (see [4] for details of CTCF-binding motif classes). (B) CTCF ChIP-seq profile for the native T39 sequence in K562 cells. The density of ChIP-seq reads are presented as vertical bars. The location of the CTCF binding motifs are indicated underneath the histogram. The putative distribution of CTCF binding is shown as shaded curves centered on each CTCF binding motif. (C) Digital DNase footprint profile for the native T39 sequence in K562 cells. The density of single-base DNase-seq reads are presented as vertical bars. The location of the CTCF binding motifs are indicated underneath the histogram.

The T39 element is located on the short arm of the X chromosome (p22.2) in the first intron of the gene ARHGAP6 (Fig 3A). The sequence of T39 contains three CTCF motifs, all from the same CTCF class 38, which exhibits an average CTCF occupancy of 90.79% [4]. Genomic profiles of the T39 sequence suggested that all three sites are bound by CTCF in K562 cells (Fig 3B), and that binding by CTCF results in distinct footprints detected by digital DNase I genomic footprinting analysis in K562 cells (Fig 3C). These properties of the T39 element appear to be ubiquitous, since strong peaks of CTCF binding and DNase hypersensitivity are seen in 19 independent cell lines [4].

### Comparison of T39 with other silencers from the literature

A partial list of silencers from the literature appears in Table 1. These silencers have been identified in cells of various species using transient reporter assays based on expression of luciferase, chloramphenicol acetyltransferase, or both. Silencer activity was uniformly assessed by transient transfection and ranged from 2-fold to 50-fold. For comparison with T39 we selected three silencers. PRE2-S5 is a silencer studied in a breast cancer cell line (MCF7) and was shown to reduce expression 50-fold compared to a no-insert control [11]. MECP2 F3 is a silencer active in several cell lines and was shown to reduce expression an average of 10-fold compared to a no-insert control [12]. PDGFA 5′ SHS is a silencer active in several cell lines and was shown to reduce expression 2- to 5-fold compared to a no-insert control [13]. When assessed using the enhancer-blocking assay described in Fig 1A, the PRE2-S5 element reduced the frequency of G418-resistant colony formation 2.4-fold compared to the neutral spacer control, while the MECP2 F3 and PDGFA 5' SHS elements had no statistically distinguishable effect on the rate of G418-resistant colony formation (Fig 2A). In comparison, the cHS4 element used as an insulator control reduced G418-resistant colony formation 2.5-fold, and the T39 silencer reduced G418-resistant colony formation 77-fold. When assessed using the silencer configuration, the PRE2 element reduced the frequency of G418-resistant colony formation 2.2-fold compared to the spacer control, while the MECP2 element had no statistically distinguishable effect on the rate of colony formation and the PDGFA element actually increased the rate of colony formation (Fig 2B). In this configuration, the cHS4 insulator had no statistically distinguishable effect on the rate of G418-resistant colony formation as expected, while the T39 silencer reduced the rate of colony formation 36-fold. Taken together, these studies indicate that PRE2-S5 functions as an autonomous silencer, while MECP2-F3 and PDGFA 5′ SHS do not function as silencers in this integration-based assay. These studies also demonstrate that the T39 element is a far more potent autonomous silencer than the PRE2-S5 element. Chromatin profiling of the PRE2, MECP2, and PDGFA native loci revealed the absence of constitutive DHSs in five independent cell lines used in their original characterizations while the native T39 loci exhibited strong DHS peaks in all five of these cell lines (S1 Fig). These profiles suggest only the T39 element should be expected to exhibit constitutive activity.

### Silencer activity is marginally influenced by context

**Fig 4:**
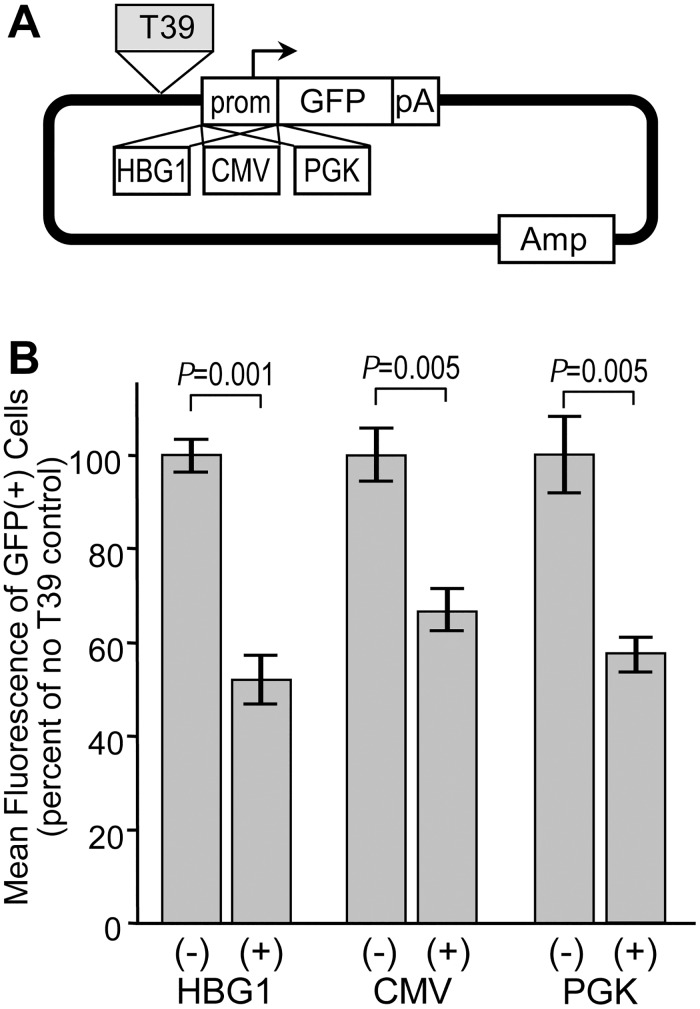
Influence of promoter on silencer activity. (A) GFP reporter construct. The reporter plasmid contains a GFP expression cassette terminated with the SV40 polyadenylation signal, linked to one of the three indicated promoters. The reporter constructs were either used directly or with the T39 silencer inserted 5′ of the promoter. (B) Assessment of reporter expression. Reporter plasmids were linearized and transfected into HEK293 cells. After 7 days, the level of reporter gene expression was analyzed by measuring the mean fluorescence of the cells expressing GFP by flow cytometry. Data are shown for the mean ± s.e. from three independent transfections, and are normalized to the mean fluorescence of the construct without the T39 silencer. P values based on one-sided *t*-test comparing constructs with and without the T39 silencer for each promoter. See S1 Table for details on datasets.

In theory, autonomous silencer elements should be capable of exerting their activity on different sources of promoters and within multiple cell lines. The G418-reisistant colony assay and associated reporter construct could not be easily used to assess these properties, since the HS2 enhancer does not function with all promoters, the HBG1 promoter does not function in all cell types, and not all cell types are capable of forming distinct colonies in agarose. For these reasons, we turned to the reporter construct diagramed in Fig 4A. This construct links various promoters to a GFP expression cassette, with candidate insulators inserted immediately 5' of the promoter. In this assay, the degree of silencing is assessed by comparing the intensity of reporter GFP expression in GFP-positive cells following stable plasmid transfection. As with the drug-resistance colony assay, this GFP reporter assay provides an opportunity to detect silencer activity in the context of stable integration and intact chromatin. However, the use of GFP provides for a very wide dynamic range that makes it possible to assess reporter gene expression from a wide range of promoters and in a wide range of cell types. Using this reporter system in easily transfectable HEK293 cells, we found that the T39 element was capable of silencing the activity of three very distinct promoters: the HBG1 promoter, a constitutive promoter from cytomegalovirus (CMV), and the constitutive promoter from the human PGK gene (Fig 4B). The degree of silencing was nearly uniform, ranging from 1.5-fold for CMV to 1.9-fold for HBG1.

**Fig 5:**
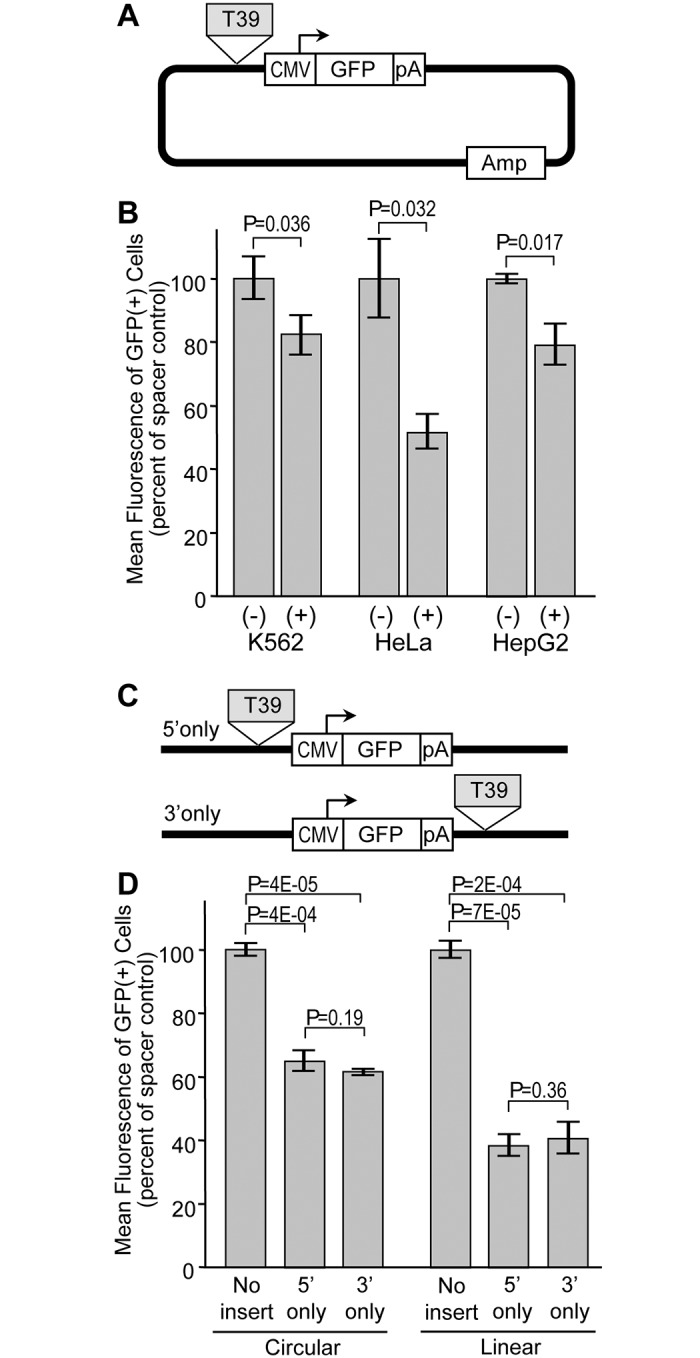
Influence of cell type and silencer topology on silencer activity. (A) GFP reporter construct for assessment in different cell lines. The reporter plasmid is the same as in Fig 3, except in this case only the constructs using the CMV promoter with and without the T39 silencer were compared. (B) Assessment of reporter expression in different cell lines. Reporter plasmids were linearized and transfected into the indicated cell lines. After 7 days, the level of reporter gene expression was analyzed by measuring the mean fluorescence of the cells expressing GFP by flow cytometry. (C) GFP reporter constructs for assessment of silencer topology. The reporter plasmids are the same as in panel A, except the T39 silencer was inserted either 5' only, or 3' only, of the CMV-GVP cassette. (D) Assessment of reporter expression for assessment of silencer topology. Reporter plasmids were left circular or were linearized and transfected into HepG2 cells. After 7 days, the level of reporter gene mean fluorescence was assessed by flow cytometry as for panel B. Data are shown for the mean ± s.e. from three independent transfections, and are normalized to the mean fluorescence of the construct without the T39 silencer. P values based on one-sided *t*-test comparing constructs with and without the T39 silencer for each cell line. See S1 Table for details on datasets.

**Fig 6:**
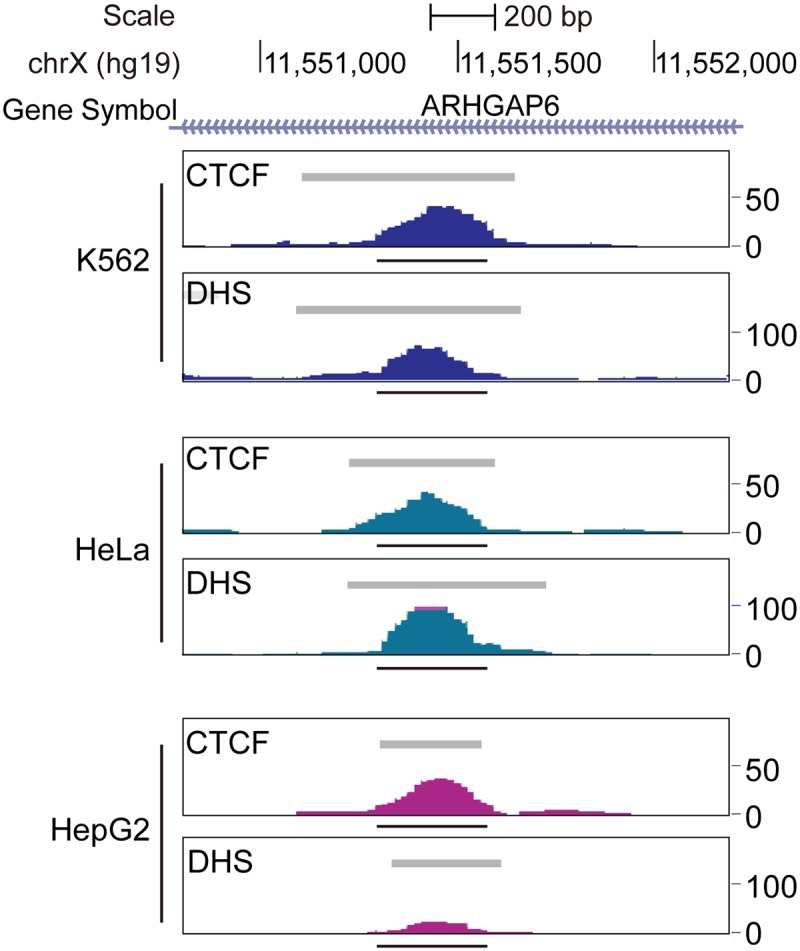
Chromatin profile of T39 in different cell lines. The raw sequence tags are shown for the CTCF-binding and DHS profiles over the 1.4 kb region centered on the native T39 sequence in the indicated cell lines. Gray bars indicate the hotspots based on peak calling. Dark horizontal bars indicate the location of the T39 sequence. T39 is located inside the first intron of ARHGAP6 (chrX:11155663–11683821, hg19).

We noted that the degree of silencing achieved with the HBG1 promoter in this setting was far less than the 36-fold observed with the same silencer and promoter in the drug-resistant colony assay. We believe this is due to the absence of the HS2 enhancer in the GFP reporter construct, and the use of a non-erythroid cell line, resulting in a substantial reduction in the basal activity of this promoter. This in turn restricts the dynamic range available for detecting the effect of a silencer. Taking this into account, we chose the reporter construct with the highly active CMV promoter for further studies (Fig 5A). As summarized in Fig 5B, we found that the T39 element silenced this promoter in erythroid K-562 cells, epithelial HeLa cells, and HepG2 liver cells. In this case the degree of silencing ranged a little more broadly, from 1.2-fold in K-562 cells to 1.9-fold in HeLa cells. In order to determine whether the silencing activity of T39 was affected by reporter construct topology, we placed this element either 5' only or 3' only of this CMV-GFP expression cassette (Fig 5C), and analyzed the effects on reporter GFP expression following transfection of HepG2 cells. As a further control, we also compared circular versus linear plasmids, since the circular topology would, in effect, place the T39 element both 5' and 3' of the CMV-GFP expression cassette. As seen in the new Fig 5D, the T39 element was equally effective at silencing expression when located in either position, with an average degree of silencing of 1.6-fold when the reporter was circular and 2.5-fold when the reporter was linear. Genomic profiling demonstrated that the T39 element is both bound by CTCF and contains a DHS in all three of the cell lines used for these studies (Fig 6).

## Discussion

The existence of transcriptional silencers has been recognized in the literature for nearly three decades (for example, see [14]). However, relatively few silencer elements have been identified and functionally characterized in mammalian cells. A survey of the literature revealed the examples summarized in Table 1. They include silencer elements derived from humans, rats, chickens, and yeast, with sizes ranging from 31 bp to 985 bp. The activities of some of these elements were assessed using the promoters and topological arrangements derived from their native loci, making it difficult to distinguish between context-dependent negative regulatory elements and context-independent autonomous silencers. The activities of other elements were assessed using heterologous promoters, enhancers, and topological arrangements in a manner that did allow an assessment for context-independent, autonomous silencer activity. Only the T39 element was studied in the context of stable gene transfer, where the reporter constructs were allowed to integrated into the target cell genome in a manner that allowed for the formation of intact chromatin; all of the other elements were studied with transient transfection assays. In addition, only the studies with the T39 element included the use of a neutral "spacer" sequence as a control, making it possible to distinguish between the direct effects of the candidate silencer element and the indirect effects of changes in spacing between enhancers, promoters, and other regulatory elements.

Of the three elements we identified from the studies of others and analyzed in the drug-resistance colony assay, only PRE2-S5 [11] exhibited autonomous silencer activity. It remains unclear why the other two elements, MECP2 F3 [12] and PDGFA 5′ SHS [13], failed to exhibit autonomous silencer activity in the drug-resistant colony assay. Both of these elements had been shown by others to function in the context of heterologous promoters, but they were not previously tested with the HBG1 promoter used in this particular assay. Likewise, neither of these elements had been previously tested in erythroid K562 cells, although they had been shown to function in several other cell lines. Perhaps most importantly, neither of these two elements had previously been tested in the context of stable gene transfer, where they would be required to function in the context of intact chromatin. Chromatin profiling suggests that these elements are not constitutively active and thus unlikely to function in a constitutive manner.

Regardless to the reasons for the discrepancies between our results and the results of others, our studies demonstrate that these two elements do not function as fully autonomous silencers.

The T39 element functioned as the most potent autonomous silencer out of the four elements analyzed here. As reported here, the T39 silencer reduced the rate of G418-resistant colony formation 77-fold in the enhancer-blocking configuration and 36-fold in the silencer configuration. Although less dramatic, this element also reduced expression of the GFP reporter constructs in the context of three distinct promoters and three distinct cell lines. In addition, this silencing activity can be achieved when T39 is used as a single copy, and when the T39 element is placed either 5' or 3' of an expression cassette. From these studies we conclude the T39 element acts as a potent, constitutive, autonomous silencer.

The T39 silencer exhibited statistically significant silencer activity in both of the drug-resistant colony assays and the GFP expression assay. However, the magnitude of this effect varied greatly between these two assay systems. Previous studies demonstrated that the drug-resistant colony assay measures the frequency of transfection events in which expression of the *neo* reporter gene exceeds a specific threshold [3]. This property allows the drug-resistant colony assay to amplify small differences in gene expression. The GFP expression assay, on the other hand, allows for a direct and linear measurement of reporter gene expression in a manner that does not amplify small differences. In addition, the drug-resistant reporter constructs contain two copies of the silencer element in a flanking arrangement, while the GFP reporter construct only contains one copy of the silencer element. This represents a difference both in the silencer dose and the ability of the silencer to block interactions between tandemly integrated reporter constructs or interactions between integrated reporter constructs and the surrounding genome.

The T39 silencer exhibits a distinct chromatin profile, including high occupancy by CTCF and DHS formation in multiple tissues. This silencer is located in the first intron of the ARHGAP6 gene. It has been suggested that silencers located in introns could suppress transcription by blocking transcriptional elongation, by preventing recognition of intronic splice sites or by abrogating basal transcriptional apparatus assembly [5,6,7]. Only the third of these mechanisms could potentially explain the silencing function of T39 in the stable transfection assays we have used. Publically available CTCF interactome data indicate that the T39 element physically interacts with other CTCF sites located at the terminus of the ARHGAP6 gene and within the neighboring gene MSL3, but not with the promoters of these genes (S2 Fig). It is of interest to speculate that the T39 silencer may modulate the expression of MSL3, a gene contributing to the X-inactivation complex of Drosophila and humans [15]. Future addition/deletion studies will be needed to specifically link CTCF binding to the silencing activity of the T39 element, as has been done in other settings [20].

In conclusion, our studies independently validated two integration-based reporter assays for the identification of silencer elements, and use these assays to validate two autonomous silencer elements: PRE2-S5 and T39. The drug-resistant colony assay provides the greatest degree of sensitivity, while the GFP reporter assay allows for a more direct assessment of silencer activity and provides a greater degree of experimental flexibility. These studies also suggest that the T39 sequence functions as a potent, constitutive autonomous silencer in a manner that is independent of the promoter or cell line. This element can be used as a positive control for studies into other silencer elements, as well as chromatin insulators. More studies are needed to dissect the mechanisms underlying silencer function, and to link these activities to specific chromatin profiles.

## Supporting Information

S1 Fig DHS profile for putative insulators in multiple cell lines. The DNase I hypersensitive site (DHS) profiles are presented for the four putative insulators in the 5 indicated cell lines. The four putative silencer elements T39, PRE2- S5, MECP2-F3, and PDGFA 5′ SH5, are described at length in the main manuscript. The five cell lines were chosen based on their use in the previous characterization of the putative insulators and the availability of publicly accessible DHS profiles. The density of DNase-seq reads are presented as vertical bars, with gray horizontal bars indicating the presence of statistically significant DHS peaks. The locations of the putative silencer sequences are indicated underneath the histograms. Each window is set at 1000 bp.
(PDF)

S2 Fig CTCF interactome for the native T39 locus. The image shows the genomic organizations of a 710 kb window around the native T39 locus in K562 cells, derived from publically available data on the UCSC Genome Browser. The location of the T39 element and three surrounding genes (ARHGAP6, AMELX, and MSL3) are indicated. Also indicated are the promoters for these genes (P). The middle two tracks show the CTCF and DHS profiles for the region. At the bottom is indicated the CTCF interactome for the region, as determined by chromatin capture technologies. Interactive regions are connected by red arches, with the intensity of the red representing the frequency of the interaction. Note that T39 interacts with a DHS/CTCF element inside the same ARHGAP6 intron, with a DHS/CTCF element at the distal end of the ARHGAP6 gene, and with a DHS/CTCF element in the intron of the adjoining MSL3 gene, but not with any of the gene promoters.
(PDF)

S1 Table Summary of Raw Data. (PDF)
