# Supplementary material for: Functional Validation of a Constitutive Autonomous Silencer Element
Source: PLoS One. 2015 Apr 24;10(4):e0124588. doi: 10.1371/journal.pone.0124588 (PMC4409358; doi:10.1371/journal.pone.0124588)
Supplement: S1 Fig — The DNase I hypersensitive site (DHS) profiles are presented for the four putative insulators in the 5 indicated cell lines. The four putative silencer elements T39, PRE2- S5, MECP2-F3, and PDGFA 5′ SH5, are described at length in the main manuscript. The five cell lines were chosen based on their use in the previous characterization of the putative insulators and the availability of publicly accessible DHS profiles. The density of DNase-seq reads are presented as vertical bars, with gray horizontal bars indicating the presence of statistically significant DHS peaks. The locations of the putative silencer sequences are indicated underneath the histograms. Each window is set at 1000 bp. (PDF) [file pone.0124588.s001.pdf]

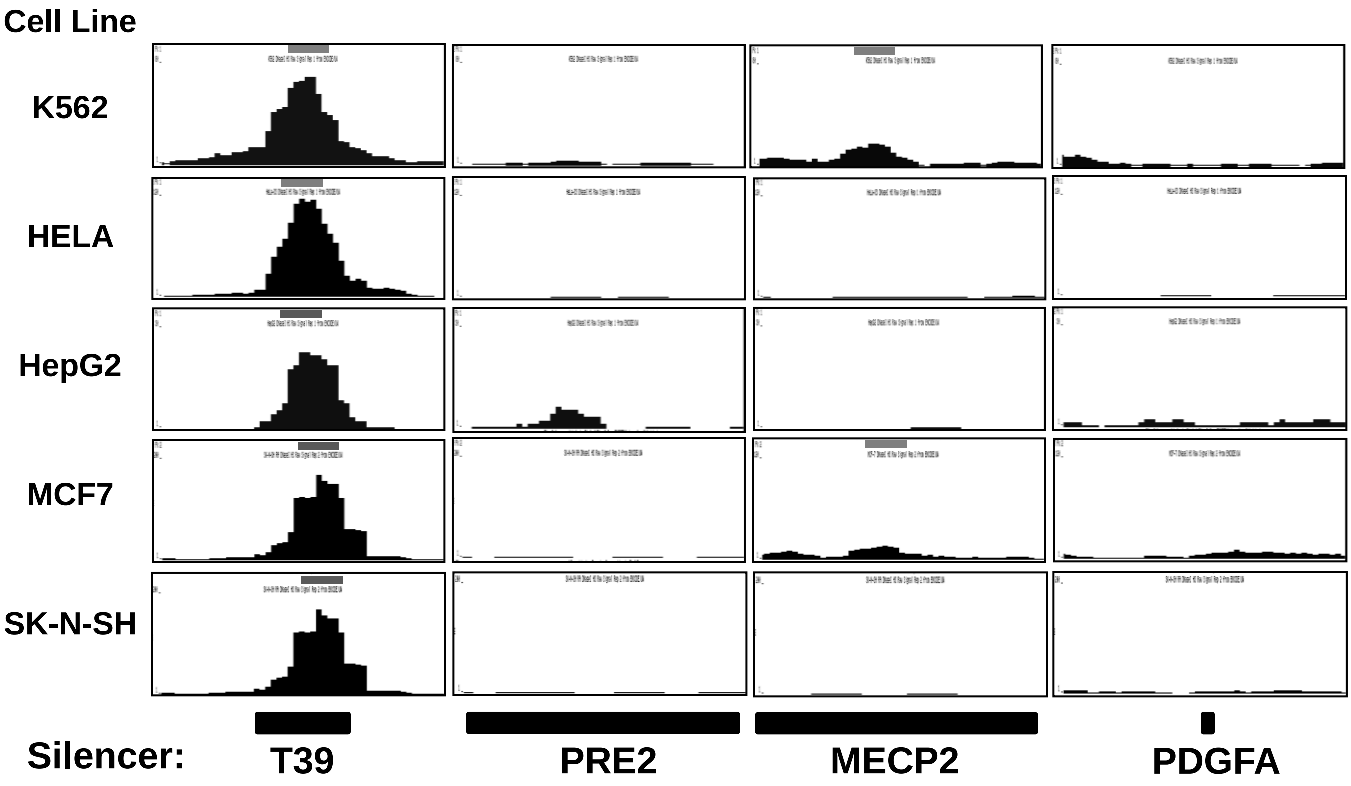

Figure S1. DHS profile for putative insulators in multiple cell lines. The DNase I hypersensitive site (DHS) profiles are presented for the four putative insulators in the 5 indicated cell lines. The four putative silencer elements T39, PRE2- S5, MECP2-F3, and PDGFA 5′ SH5, are described at length in the main manuscript. The five cell lines were chosen based on their use in the previous characterization of the putative insulators and the availability of publicly accessible DHS profiles. The density of DNase-seq reads are presented as vertical bars, with gray horizontal bars indicating the presence of statistically significant DHS peaks. The locations of the putative silencer sequences are indicated underneath the histograms. Each window is set at 1000 bp.
