# Supplementary material for: Functional Validation of a Constitutive Autonomous Silencer Element
Source: PLoS One. 2015 Apr 24;10(4):e0124588. doi: 10.1371/journal.pone.0124588 (PMC4409358; doi:10.1371/journal.pone.0124588)
Supplement: S2 Fig — The image shows the genomic organizations of a 710 kb window around the native T39 locus in K562 cells, derived from publically available data on the UCSC Genome Browser. The location of the T39 element and three surrounding genes (ARHGAP6, AMELX, and MSL3) are indicated. Also indicated are the promoters for these genes (P). The middle two tracks show the CTCF and DHS profiles for the region. At the bottom is indicated the CTCF interactome for the region, as determined by chromatin capture technologies. Interactive regions are connected by red arches, with the intensity of the red representing the frequency of the interaction. Note that T39 interacts with a DHS/CTCF element inside the same ARHGAP6 intron, with a DHS/CTCF element at the distal end of the ARHGAP6 gene, and with a DHS/CTCF element in the intron of the adjoining MSL3 gene, but not with any of the gene promoters. (PDF) [file pone.0124588.s002.pdf]

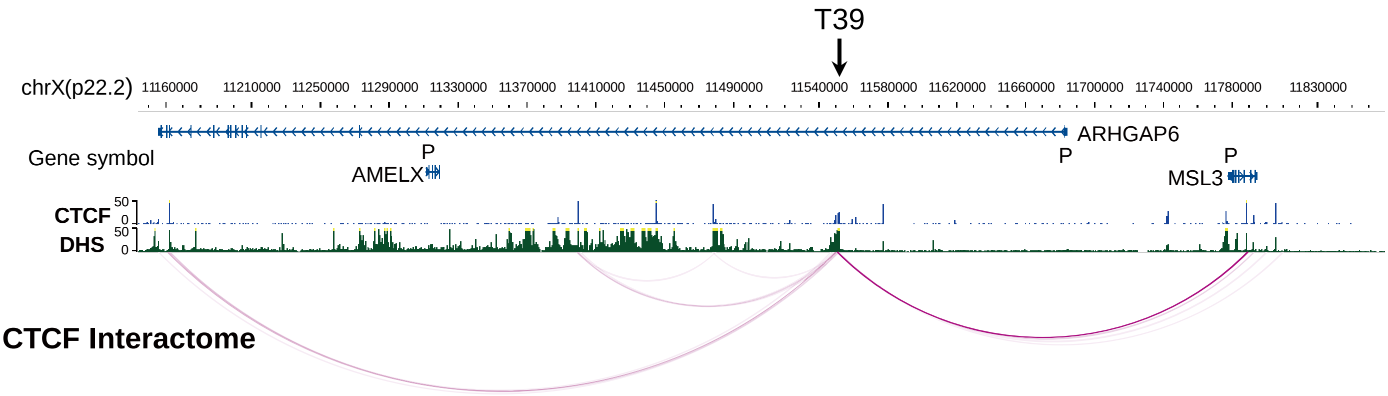


**Figure S2. CTCF interactome for the native T39 locus.** The image shows the genomic organizations of a 710 kb window around the native T39 locus in K562 cells, derived from publically available data on the UCSC Genome Browser. The location of the T39 element and three surrounding genes (ARHGAP6, AMELX, and MSL3) are indicated. Also indicated are the promoters for these genes (P). The middle two tracks show the CTCF and DHS profiles for the region. At the bottom is indicated the CTCF interactome for the region, as determined by chromatin capture technologies. Interactive regions are connected by red arches, with the intensity of the red representing the frequency of the interaction. Note that T39 interacts with a DHS/CTCF element inside the same ARHGAP6 intron, with a DHS/CTCF element at the distal end of the ARHGAP6 gene, and with a DHS/CTCF element in the intron of the adjoining MSL3 gene, but not with any of the gene promoters.
