# Supplementary material for: Functional Validation of a Constitutive Autonomous Silencer Element
Source: PLoS One. 2015 Apr 24;10(4):e0124588. doi: 10.1371/journal.pone.0124588 (PMC4409358; doi:10.1371/journal.pone.0124588)
Supplement: S1 Table — (PDF) [file pone.0124588.s003.pdf]

Table S1 - Summary of Raw Data

Figure 2A - Enhancer-Blocking Colony Assay

				Raw Count			Normalized		
	No. Exps	No. Txfs	No. Dishes	Avg	S.E.	Range	Avg	S.E.	Range
Spacer	3	10	30	94.53	8.13	20 to 180	1.00	0.05	0.57 to 1.64
cHS4	3	10	30	36.03	4.13	3 to 80	0.39	0.05	0.09 to 0.85
T39	1	4	12	1.25	0.25	0 to 2	0.01	0.003	0.00 to 0.02
PRE2	1	2	6	13.33	3.77	2 to 26	0.35	0.12	0.06 to 0.80
MECP2	1	2	6	32.00	5.30	7 to 42	0.92	0.17	0.22 to 1.33
PDGFA	1	2	6	23.17	4.56	10 to 43	0.62	0.17	0.31 to 1.33

Figure 2B - Silencer Assay Colony Assay

				Raw Count			Normalized		
	No. Exps	No. Txfs	No. Dishes	Avg	S.E.	Range	Avg	S.E.	Range
Spacer	1	2	6	24.67	4.86	7 to 40	0.76	0.15	0.22 to 1.24
cHS4	1	2	6	21.50	4.45	11 to 36	0.62	0.14	0.34 to 1.11
T39	1	4	12	3.50	0.31	2 to 5	0.03	0.003	0.02 to 0.04
PRE2	1	2	6	11.17	3.34	1 to 24	0.30	0.11	0.03 to 0.74
PDGFA	1	2	6	56.33	3.81	47 to 69	1.49	0.29	1.45 to 2.13
MECP2	1	2	6	27.50	4.81	14 to 41	0.78	0.16	0.43 to 1.27

Figure 4 - Promoter Comparison GFP Assay

				Raw Mean Fluorescene			Normalized		
	No. Exps	No. Txfs	No. Wells	Avg	S.E.	Range	Avg	S.E.	Range
HBG1(-T39)	1	3	3	1238	42	1159 to 1302	1.00	0.03	0.94 to 1.05
HBG1(+T39)	1	3	3	646	66	554 to 773	0.52	0.05	0.45 to 0.62
CMV(-T39)	1	3	3	50962	2929	47764 to 56812	1.00	0.06	0.94 to 1.11
CMV(+T39)	1	3	3	33978	2293	31447 to 38556	0.67	0.05	0.62 to 0.76
PGK(-T39)	1	3	3	5648	471	4811 to 6442	1.00	0.08	0.85 to 1.14
PGK(+T39)	1	3	3	3243	211	2839 to 3553	0.57	0.04	0.5 to 0.63

Figure 5B - Cell Type Comparison GFP Assay

				Raw Mean Fluorescene			Normalized		
	No. Exps	No. Txfs	No. Wells	Avg	S.E.	Range	Avg	S.E.	Range
K562(-T39)	1	3	3	3372	125	3222 to 3621	1.00	0.07	0.96 to 1.07
K562(+T39)	1	3	3	2779	209	2381 to 3807	0.82	0.06	0.71 to 0.92
HeLa(-T39)	1	3	3	4860	613	3876 to 5986	1.00	0.13	0.80 to 1.23
HeLa(+T39)	1	3	3	2510	269	2241 to 2778	0.52	0.06	0.46 to 0.57
HepG2(-T39)	1	3	3	1420	14	1400 to 1446	1.00	0.01	0.99 to 1.02
HepG2(+T39)	1	3	3	1121	93	997 to 1304	0.79	0.07	0.70 to 0.92

Figure 5D - 5' Versus 3' Comparison GFP Assay

				Raw Mean Fluorescene			Normalized		
	No. Exps	No. Txfs	No. Wells	Avg	S.E.	Range	Avg	S.E.	Range
Circular									
No insert	1	3	3	5273	109	5108 to 5478	1.00	0.02	0.97 to 1.04
T39 5' only	1	3	3	3415	166	3185 to 3737	0.65	0.03	0.60 to 0.71
T39 3' only	1	3	3	3241	56	3132 to 3314	0.62	0.01	0.59 to 0.63
Linear									
No insert	1	3	3	4600	121	4376 to 4791	1.00	0.03	0.95 to 1.04
T39 5' only	1	3	3	1769	158	1490 to 2037	0.38	0.03	0.32 to 0.44
T39 3' only	1	3	3	1874	226	1524 to 2295	0.41	0.05	0.33 to 0.50

No. Exps:	Number of independent experiments
No. Txfs:	Number of independent transfections
No. Dishes:	Number of independent dishes plated and scored by counting colonies under G418 selectioin
No. Wells:	Number of independent wells plated and scored for fluorescence by flow cytometry
Avg:	Average
S.E.:	Standard Error
Range:	Range of values (lowest and hightest)
Raw Count:	Number of colonies per dish (Figures 2A and 2B)
Raw Mean Fluorescence:	Mean Fluorescene units per sample (Figures 4, 5B, 5D)
